# MiR-186-3p attenuates tumorigenesis of cervical cancer by targeting IGF1

**DOI:** 10.1186/s12957-021-02317-z

**Published:** 2021-07-12

**Authors:** Xiurong Lu, Xiao Song, Xiaohui Hao, Xiaoyu Liu, Xianyu Zhang, Na Yuan, Huan Ma, Zhilin Zhang

**Affiliations:** Department of Radiotherapy, The First Affiliated Hospital of Hebei North University, No. 36, Changqing Road, Zhangjiakou, 075000 Hebei China

**Keywords:** Cervical cancer, miR-186-3p, IGF1, Proliferation, Migration, Apoptosis

## Abstract

**Background:**

Mounting evidence in the cancer literature suggests that microRNAs (miRNAs) influence the progression of human cancer cells by targeting protein-coding genes. How insulin-like growth factor 1(IGF1) and miR-186-3p contribute to the development of cervical cancer (CC) remains unclear. This study examined the regulatory roles of miR-186-3p and IGF1 in CC development.

**Methods:**

Gene expression levels were determined by qRT-PCR. Proliferation, migration, and apoptosis of CC and normal cells were determined by MTT, Transwell, and caspase-3 activity assays, respectively. Dual-luciferase reporter activity and RNA pull-down assays were performed to identify the target gene of miR-186-3p.

**Results:**

IGF1 was the target of miR-186-3p. The expression of miR-186-3p inhibited cell proliferation and migration abilities of CC cell lines, but induced the apoptosis rate of CC cells. IGF1 could restore the inhibitory effects of miR-186-3p on the proliferation, migration, and apoptosis abilities of CC cells. Experimental results revealed that miR-186-3p could inhibit IGF1 expression, thereby reducing the viability of CC cells.

**Conclusions:**

The data suggest that targeting of IGF1 by miR-186-3p could be crucial in regulating the progression of CC.

## Background

Cervical cancer (CC) is a global threat with high mortality and incidence rates, and low survival rate [1]. Most CC cases are caused by human papillomavirus (HPV) infection. HPV screening and vaccination can effectively prevent CC. Present-day CC treatments depend on a clinical staging system. The standard clinical treatment for patients with early stage CC is hysterectomy, lymphadenectomy, chemotherapy, or radiation therapy [2–4]. Patients at the advanced stage of CC are usually treated with brachytherapy [5]. However, these clinical treatment approaches are ineffective in preventing CC recurrence [6]. Hence, there is an urgent need to understand the underlying molecular mechanisms of CC initiation and progression.

MicroRNAs (miRNAs) are small non-coding RNA molecules that bind to the 3'-untranslated region (UTR) of target mRNAs and regulate the translation and expression of genes [7]. By changing the bioactivity of cancer-promoting or tumor-suppressing genes, miRNAs suppress or facilitate the progression of various cancer types, including CC [8]. Previous studies have indicated that the degradation or repression of CC could be influenced by various miRNAs, such as miR-21, miR-29a, miR-451a, and miR-106b-5p [9, 10]. A growing body of research has also demonstrated that miR-186 participates in several cellular processes, including cell proliferation, migration, apoptosis, and the cell cycle [11–15]. For instance, miR-186 downregulates CC tumors and contributes to tumor growth [16, 17]. miR-186 also can promote or suppress CC cell development [16, 17]. However, the role of miR-186-3p in CC has not yet been explored.

The insulin-like growth factor 1 (IGF1) gene is located on chromosome 12q23.2. The gene comprises seven exons and encodes a protein that is functionally and structurally similar to the insulin involved in cell growth and development [18]. Accumulating evidence in the past few decades has revealed that IGF1 is abnormally expressed in various cancer types [19–22]. However, only a few studies have shown the upregulation of IGF1 in CC cells [23–25]. The molecular mechanism of the interaction of miR-186-3p with IGF1 during the progression of CC is still unknown.

This study investigated the regulatory roles of miR-186-3p and IGF1 in CC development. We explored the underlying mechanism of miR-186-3p and IGFI in CC progression. The study hypothesis was that by targeting IGF1, miR-186-3p might inhibit the development of CC. The data provide insights into CC progression, treatment, and diagnosis.

## Materials and methods

### Clinical tissue samples

CC tissue samples and adjacent normal tissues were obtained from 35 patients. The collection was performed with the approval of the ethics committee of our hospital. All participants consented to participate in the survey. The samples collected from patients were stored in liquid nitrogen until analysis. Table 1 shows patient characteristics.

**Table 1:** Clinical characteristics of patients with cervical cancer

Characteristic	Number	Proportion(%)
Total	35	
Age (years)		
< 45	12	34.29
≥ 45	23	65.71
Depth of cervical stromal invasion		
≤ 1/2	9	25.71
> 1/2	26	74.29
LVSI		
Negative	11	31.43
Positive	24	68.57
Tumor size (cm)		
≤ 4	19	54.29
> 4	16	45.71
Histologic diagnosis		
Adenocarcinoma	3	8.57
Squamous carcinoma	30	85.71
Others	2	5.71
FIGO stage		
IB	22	62.86
IIA	13	37.14
Neoadjuvant chemotherapy		
Yes	14	40.00
No	12	34.29
Unknown	9	25.71

*LVSI* lymphovascular space invasion, *FIGO* International Federation of Gynecology and Obstetrics

### Cell culture and transfection

Normal cervical cells (HcerEpic) and three human CC cell lines (HeLa, CaSKi, SiHa, and C33A) were purchased from ATCC (USA). The HcerEpic cell line was cultured in Dulbecco’s Minimum Essential Medium (DMEM). The CaSKi cell line was cultured in RPMI-1640 medium. The HeLa, SiHa, and C33A cell lines were cultured in Eagle’s Minimum Essential Medium. All cells were cultured with 10% fetal bovine serum (Gibco, USA), 100 U/mL penicillin, and 100 μg/mL streptomycin (cat# 15070063, Thermo Fisher Scientific, USA) in a humidified atmosphere containing 5% CO_2_. HeLa or SiHa cells (2 × 10^5^) were inoculated into a 6-well plate and incubated for 24 h. The cells were then transfected with Lipofectamine 3000 (Thermo Fisher Scientific). The inhibitors and mimics of miR-186-3p were purchased from Active Motif, Inc. (USA).

### RNA extraction

The miRNeasy FFPE Kit (50) (cat# 217504, Qiagen, Germany) was used to extract mRNA from tissues. The extraction was performed according to the manufacturer’s instructions. HcerEpic, HeLa, Ca SKi, SiHa, and C33A cells (6 × 10^5^) were collected, and total RNA was obtained using TRIzol Reagent (Thermo Fisher Scientific).

### Quantitative real-time polymerase chain reaction (qRT-PCR)

RNA was reverse-transcribed using the Reverse Transcription Kit (Cat # 18090010, Invitrogen, USA). The performance of qRT-PCR was then examined with SYBR® Premix Ex Taq (cat# RR820A, TaKaRa Bio, Japan) using the 7300 Real-Time PCR System (Applied Biosystems, USA). The ΔΔCt method was used for relative quantification. Glyceraldehyde 3-phosphate dehydrogenase (GAPDH) or U6 was used as the reference gene. The primers used are shown in Table 2.

**Table 2:** The sequences of the primers in this study

Primer	Sequences
**IGF1**	Forward: 5'-TCGCATCTCTTCTATCTGGCCCTGT-3'
	Reverse: 5'-GCAGTACATCTCCAGCCTCCTCAGA-3'
**GAPDH**	Forward: 5'-CAGCCTCAAGATCATCAGCA-3'
	Reverse: 5'-GGCATGGACTGTGGTCATGAG-3'
**U6**	Forward: 5'-CTCGCTTCGGCACA-3'
	Reverse: 5'-AACGCTTCACGAATTTGCGT-3'

### Cell viability assessment

Cell viability was examined using the 3-(4,5-dimethylthiazol-2-yl)-2,5-diphenyltetrazolium bromide (MTT) Cell Viability Assay Kit (cat# AR1156, Boster Biological Technology, USA). The cells were cultured and incubated for 4 h at 37 °C after the addition of MTT solution (5 mg/mL). The optical density at 570 nm was determined using a microplate reader (Thermo Fisher Scientific).

### BrdU cell proliferation assessment

This assessment was performed using the BrdU Cell Proliferation Assay Kit (cat# 6831, Cell Signaling Technology, USA). The cells were seeded in 96-well plates (5 × 10^4^ cells per well). A 1 × BrdU solution was added to each well. The mixture was incubated for 4 h. After removing the medium, fixing/denaturing solution was added, and the cells were incubated at 25 °C for 30 min. Then, both 1× detection antibody solution and 1 × horseradish peroxidase (HRP) labeled secondary antibody solution were added to the cells, and the mixture was incubated with 3,3′,5,5′-tetramethylbenzidine (TMB) substrate for 30 min. After 100 μL of STOP solution were added, the absorbance at 450 nm was measured using a microplate reader.

### Cell migration assays

The cells (5 × 10^4^ cells/well) were collected and seeded in 96-well plates. After that, they were cultured in the upper chambers of a Transwell device in serum-free medium (200 μL). Next, 600 μL DMEM with 10% serum were added to the lower chambers of the Transwell device. After incubation for 24 h, the cells that failed to migrate were removed with a cotton swab, while those that migrated were fixed with fixing solution (4% v/v formaldehyde in PBS). Subsequently, the cells were stained with 0.1% crystal violet for 30 min. Images of the cells were captured using the Evos FL Auto Cell Imaging System (Thermo Fisher Scientific).

### Cell apoptosis assays

Caspase-3 activity was measured using a Human Active Caspase-3 Ser29 ELISA Kit (Cat# ab181418, Abcam, UK). Fifty microliters of standard or sample were added to the wells. An antibody cocktail (50 μL) was added for 1 h. TMB Development Solution (100 μL) was added for 10 min. Stop solution (100 μL) was added and the absorbance was read at 450 nm.

### Western blotting assay

Total protein from cells was isolated using RIPA buffer (Sigma-Aldrich, USA). The protein concentration was determined using a BCA protein assay kit (BioRad, USA). The protein constituents in 20 μg of total protein were separated by 12% SDS-PAGE. The proteins were transferred to polyvinylidene fluoride membranes. The membranes were blocked with 5% non-fat milk for 1 h at room temperature and incubated with primary antibodies, including Caspase-3 (Cat# ab184787, Abcam) and GAPDH (Cat# ab9485, Abcam) overnight at 4 °C. The next day, the membranes were incubated with the secondary antibody (Cat#: ab6721, Abcam, USA) at room temperature for 1 h. Finally, the protein bands were visualized using a chemiluminescent substrate (Thermo Fisher Scientific).

### Luciferase activity assay

Wild-type or mutant IGF1 was synthesized and cloned into the pGL3/Luciferase (Luc) vector. MiR-186-3p mimic or negative control (NC) mimic was co-transformed into HeLa cells or SiHa cells with wild-type or mutant IGF1. After 48 h of transfection, cells were treated with RIPA buffer. A dual-luciferase assay system (Promega Corporation, USA) was used to detect luciferase activity.

### RNA pull-down assay

MiR-186-3p and NC were biotin-labeled using a biotin RNA labeling mix and T7/SP6 RNA polymerase. The RNeasy Mini Kit (cat# 74104, Qiagen) was used to verify IGF1 expression by qRT-PCR.

### Statistical analyses

All statistical analyses were performed using SPSS software (version 19.0; IBM SPSS, USA). Three repeats were performed in each experiment. The data are presented as the mean ± standard deviation. The differences between two groups were measured using Student's *t* test. The differences among multiple groups were measured using analysis of variance (ANOVA). Statistical significance was set at *P* < 0.05.

## Results

### MiR-186-3p inhibits development of CC cells

**Fig. 1:**
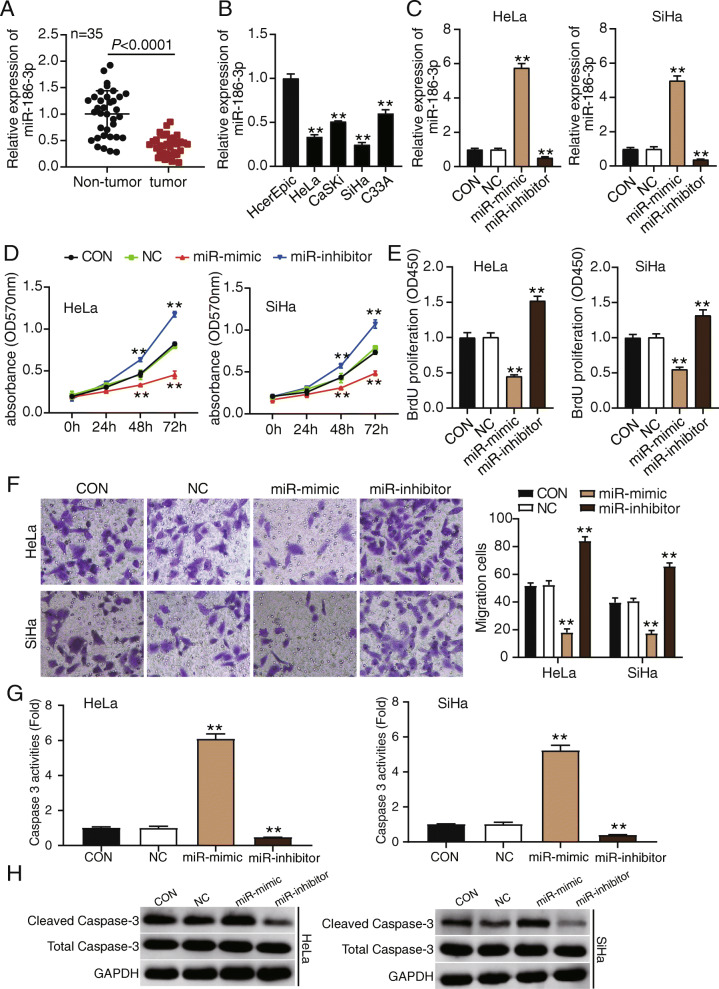
MiR-186-3p inhibits the development of cervical cancer cells. **A** qRT-PCR detection of the relative expression of miR-186-3p in non-tumor tissues and tumor tissues. *P* < 0.001 using Student’s *t* test. **B** qRT-PCR detection of the relative expression of miR-186 in HcerEpic, HeLa, CaSKi, SiHa, and C33A cells. ^**^ *P* < 0.001 compared with HcerEpic cells using ANOVA. **C** qRT-PCR detection of the relative expression of miR-186-3p in HeLa and SiHa cells transfected with miR-186-3p mimic and inhibitor and NC. **D** MTT assay detection of the viability of HeLa and SiHa cells transfected with miR-186-3p mimic and inhibitor and NC. **E** BrdU assay determination of the proliferation of HeLa and SiHa cells transfected with miR-186-3p mimic and inhibitor and NC. **F** Transwell assay measurement of the migration ability of HeLa and SiHa cells transfected with miR-186-3p mimic and inhibitor and NC. **G** Caspase-3 activity assay detection of cell apoptosis of HeLa and SiHa cells transfected with miR-186-3p mimic and inhibitor and NC. **H** Western blotting detection of the expression of cleaved caspase-3 and total caspase-3 in HeLa and SiHa cells transfected with miR-186-3p mimic and inhibitor and NC. **C**–**H** ^**^, *P* < 0.001 compared with blank control (CON) group using ANOVA. Other abbreviations: *NC*, negative control; *miR-mimic*, miR-186-3p mimic; *miR-inhibitor*, miR-186-3p inhibitor

We collected 35 paired CC tissue samples and adjacent normal tissues to examine the miR-186-3p expression levels. MiR-186-3p expression in tumor tissues was 60% lower than that in normal tissues (Fig. 1A). MiR-186-3p expression levels were further verified in HeLa, CaSKi, SiHa, and C33A CC cells. A 50% decline in miR-186-3p expression was evident in CC cell lines, compared with the expression in the HcerEpic normal cervical epithelial cell line (Fig. 1B). HeLa and SiHa cells were selected for further analyses because their miR-186-3p expression level was the lowest, compared with that of other CC cells. MiR-186-3p expression in miR-186-3p mimics was five times more than that in the control group. MiR-186-3p expression in the miR-186-3p inhibitor was 0.6 times lower than that in the control group (Fig. 1C).

The functional effects of miR-186-3p mimics and miR-186-3p inhibitors on CC cells were assessed. MTT assay data indicated that miR-186-3p mimics inhibited cell viability and that transfection of miR-186-3p inhibitor increased cell viability (Fig. 1D). The MTT assays results were similar to those of the BrdU assays. In the latter, miR-186-3p mimics inhibited cell proliferation by 50%, compared with the control group. Cell proliferation of the miR-186-3p inhibitor group was 1.5-fold higher than that of the control group (Fig. 1E). After transfection with miR-186-3p mimics, cell migration was reduced by 50% compared with that of the control group. However, cell migration increased after transfection with the miR-186-3p inhibitor (Fig. 1F). Finally, cell apoptosis was examined by measuring caspase-3 activity. The caspase-3 activity of the miR-186-3p mimic group was 6-fold higher than that of the control group but was reduced by 70% after transfection with the miR-186-3p inhibitor (Fig. 1G). The western blotting assay also proved that the miR-186-3p mimic enhanced the expression of cleaved caspase-3, whereas the miR-186-3p inhibitor inhibited cleaved caspase-3 expression (Fig. 1H). These results suggest that miR-186-3p can restrain the growth and development of CC cells.

### Identification of IGF1 as the key gene of interest

**Fig. 2:**
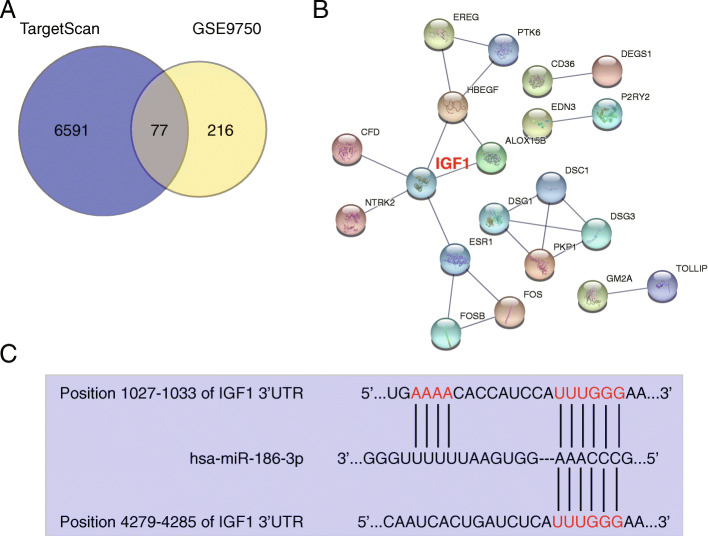
Prediction of IGF1 gene as the key gene and the target of miR-186-3p in cervical cancer. **A** Seventy-seven upregulated genes overlapped in TargetScan and GSE9750. TargetScan is an online tool to predict the target genes of miR-186-3p. GSE9750 is a mRNA microarray to screen the upregulated genes in cervical cancer with adjusted *P* < 0.05 and log_2_FC > 2. **B** The protein–protein interactions of 77 upregulated genes were constructed by STRING. **C** The binding sites between IGF1 and miR-186-3p were predicted by TargetScan

TargetScan was used to predict the miR-186-3p target genes. The GSE9750 mRNA microarray from GEO DataSets was used to screen the upregulated genes in CC with an adjusted *p* < 0.05 and log_2_FC > 2. Venny 2.1.0 analysis revealed a total of 77 upregulated genes that overlapped with the TargetScan and GSE9750 databases (Fig. 2A). These overlapping genes were subsequently uploaded to STRING to observe the protein–protein interactions. IGF1 was identified as the central gene because its encoded protein was connected with five other proteins (Fig. 2B). Subsequent CC experiments focused on IGF1. Moreover, TargetScan predicted that the IGF1 3' untranslated repeat (UTR) had two binding sites for miR-186-3p (Fig. 2C). The relationship between IGF1 and miR-186-3p was further explored.

### MiR-186-3p directly targets and inhibits IGF1

**Fig. 3:**
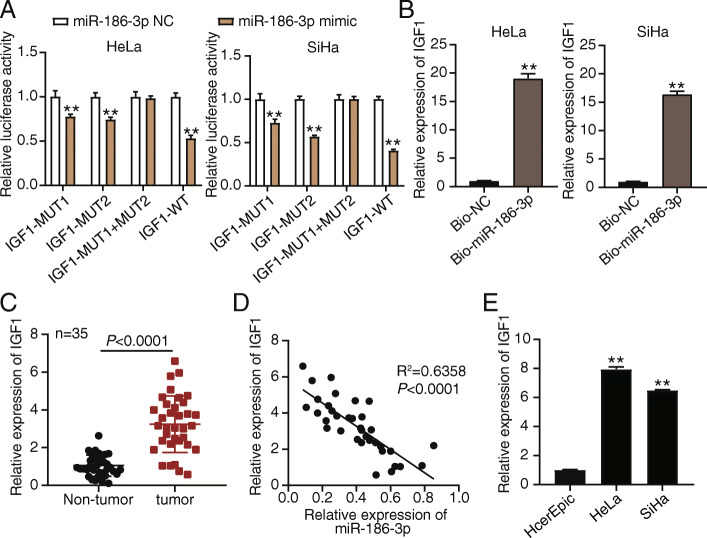
MiR-186-3p directly targets IGF1. **A** Relative luciferase activity of the indicated IGF1 reporter construct in HeLa and SiHa cells is shown. ^**^ *P* < 0.001 compared with miR-186-3p NC group using ANOVA. **B** RNA pull-down experiment showed that the biotinylated miR-186-3p in HeLa and SiHa successfully pulled down IGF1. ^**^ *P* < 0.001 using Student’s *t* test. **C** The mRNA expression level of IGF1 in non-tumor or tumor tissue was detected by qRT-PCR. *P* < 0.001 using Student’s *t* test. **D** Pearson correlation analysis between the expression level of miR-186-3p and IGF1. **E** The relative expression of IGF1 in HcerEpic, HeLa, and CaSKi cells was measured by qRT-PCR. ^**^ *P* < 0.001 compared with HcerEpic cells using ANOVA. *NC*, negative control; *WT*, wild-type; *MUT*, mutant

Based on the results of bioinformatics analysis, we designed a mutant of IGF1 that cannot bind to miR-186-3p. In HeLa and SiHa cells co-transfected with miR-186-3p mimics, the relative luciferase activity of the IGF1 wild-type was reduced by half, compared with the control group. Nonetheless, the luciferase activity of the mutant-IGF1-3'UTR reporter gene was not affected (Fig. 3A). RNA pull-down experiments revealed that biotinylated miR-186-3p could pull down IGF1 in HeLa and SiHa cells (Fig. 3B). IGF1 expression in tumor tissues was 3-fold higher than that in normal tissues (Fig. 3C). Moreover, miR-186-3p expression was negatively correlated with IGF1 expression in tumor tissues (Fig. 3D). Comparison of IGF1 expression between normal cervical cells and CC cells, revealed that the relative expression of IGF1 in HeLa and CaSKi cells was 6-fold higher than that in the HcerEpic cells (Fig. 3E). These data suggest that IGF1 could be highly expressed in cancer tissues and that miR-186-3p could bind to the 3'UTR of IGF1 and inhibit IGF1 expression.

### IGF1 knockdown eliminates the effect of miR-186-3p inhibitor

**Fig. 4:**
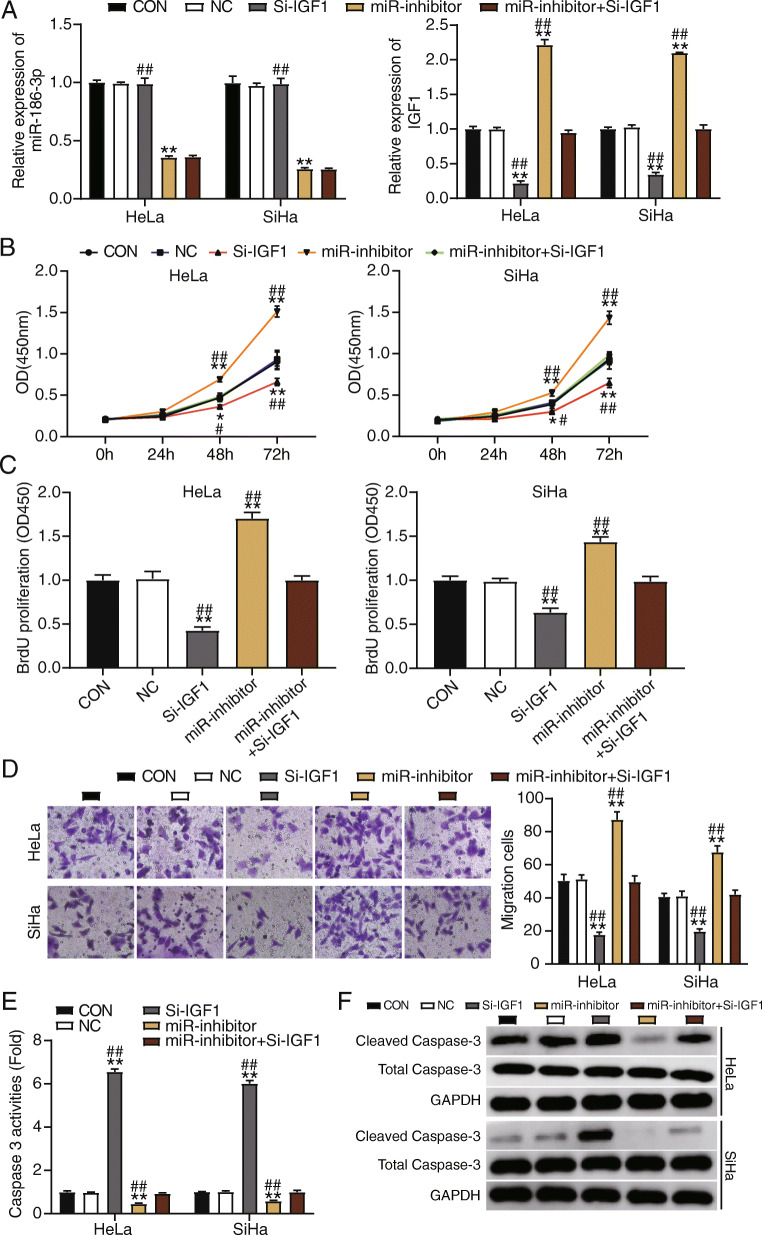
IGF1 and miR-186-3p have opposite effects in HeLa and SiHa cells. **A** qRT-PCR detection of the relative expression levels of miR-186-3p and IGF1 in HeLa and SiHa cells transfected with Si-IGF1, inhibitor and inhibitor + Si-IGF1. **B** MTT assay determination of viability of HeLa and SiHa cells transfected with Si-IGF1, inhibitor and inhibitor + small interfering-(Si) IGF1. **C** Cell proliferation of HeLa and SiHa by BrdU assay was determined in HeLa and SiHa cells transfected with Si-IGF1, inhibitor and inhibitor + Si-IGF1. **D** Transwell assay measurement of migration of HeLa and SiHa cells transfected with Si-IGF1, inhibitor and inhibitor + Si-IGF1. **E** Detection of caspase-3 activity of HeLa and SiHa cells transfected with Si-IGF1, inhibitor and inhibitor + Si-IGF1. **F** Western blotting detected the expression of cleaved caspase-3 and total caspase-3 in HeLa and SiHa cells transfected with Si-IGF1, inhibitor and inhibitor + Si-IGF1. ^*^ *P* < 0.05; ^**^ *P* < 0.001 compared with CON group using ANOVA. ^#^ *P* < 0.05; ^##^ *P* < 0.001 compared with inhibitor + Si-IGF1 group using ANOVA. *CON*, blank control. *NC*, negative control; *Si-IGF1*, SiRNA-IGF1; *inhibitor*, miR-186-3p inhibitor

To investigate the impact of IGF1 and miR-186-3p on CC cell proliferation and metastasis, we explored the relationship between miR-186-3p and IGF1. After knockdown of IGF1, the levels of miR-186-3p in HeLa and SiHa cells were consistent with those in the control group. MiR-186-3p expression in the miR-186-3p inhibitor decreased by 60%. The same result was observed after co-transfection with small interfering IGF1 and miR-186-3p inhibitor. Evaluation of the expression levels of IGF1 showed that the relative expression level of IGF1 was twice that of the control group after transfecting with the miR-186-3p inhibitor, while IGF1 expression in the cells transfected with Si-IGF1 decreased by 60% compared to that in control cells. After knocking down IGF1 and transfecting the miR-186-3p inhibitor, IGF1 expression returned to the same level as that in the control group (Fig. 4A).

In the MTT assay, cell proliferation decreased when IGF1 was knocked down, and the cell activity increased after transfection with miR-186-3p inhibitor. However, the cell activity was the same as that of the control group when IGF1 was knocked down and the miR-186-3p inhibitor was transfected simultaneously (Fig. 4B). BrdU assay results similarly indicated that compared with the control group, IGF1 knockdown decreased the cell proliferation level, and the miR-186-3p inhibitor accelerated the cell proliferation level by 1.5-fold. However, the level of cell proliferation was the same as that in the control group when IGF1 was knocked down and the miR-186-3p inhibitor was transfected simultaneously (Fig. 4C).

We further examined the effects of IGF1 and miR-186-3p on the migration ability of HeLa and SiHa cells. When IGF1 was knocked down, the number of migrating cells was reduced by half compared to that in the control group, and cell migration increased significantly after cells were transfected with the miR-186-3p inhibitor. However, when IGF1 was knocked down and the miR-186-3p inhibitor was transfected simultaneously, the number of migrating cells was similar to that in the control group (Fig. 4D). Finally, we performed caspase-3 activity analysis to verify the apoptosis of HeLa and SiHa cells. Caspase-3 activity decreased after cells were transfected with the miR-186-3p inhibitor. We also noticed that caspase-3 activity significantly increased by 6-fold, compared with the control group after IGF1 knockdown. However, the caspase-3 activity of the cells returned to the level of the control group after knockdown of IGF1 and transfection with the miR-186-3p inhibitor (Fig. 4E). The cleaved caspase-3 protein was enhanced after knockdown of IGF1, and co-transfection with si-IGF1 and miR-186-3p inhibitor relieved the promotive effect of si-IGF1 on cleaved caspase-3 protein (Fig. 4F). The collective results indicate that IGF1 knockdown could eliminate the effect of the miR-186-3p inhibitor in CC cells.

## Discussion

Although numerous studies have documented the tumorigenic effects of miRNAs on CC cells, the molecular mechanisms underlying these effects are still unclear. The biological function of miR-186 in cancer involves different cellular processes, including proliferation, migration, apoptosis, and cell cycle regulation [26]. MiR-186 can downregulate various cancer types, such as acute myeloid leukemia, non-small cell lung cancer, oral squamous cell carcinoma, and hepatocellular carcinoma [27–30]. In one study, miR-186-3p restrained the development of breast tumors [31]. In another study, miR-186-3p reduced the expression of cyclin-dependent kinase 1 (CDK1) and influenced the cell cycle regulation of cancer cells [32]. However, the characteristics of miR-186-3p in CC and its relationship with CC development are unclear. The present study is the first examination of the regulatory roles of miR-186-3p and IGF1 in CC development.

We hypothesized that miR-186-3p inhibits IGF1 and regulates the proliferation and metastasis of CC cells. To confirm this hypothesis, we performed bioinformatics analysis to identify the target gene (IGF1) for miR-186-3p. Previous studies confirmed the vital role of IGF1 in the development of cancer by regulating cell proliferation and apoptosis [33]. IGF1 participates in the growth and invasiveness of breast cancer [34, 35]. IGF1 expression regulates the proliferation, migration, and invasion abilities of CC cells [36, 37]. The results of our research are consistent with the results of these studies. We demonstrated the importance of IGF1 in the development of CC cells, and showed that miR-186-3p can target IGF1. The data suggest that miR-186-3p could target and regulate the function of IGF.

Furthermore, by targeting IGF1 and inhibiting its expression, miR-186-3p decreased the proliferation and migration abilities of CC cells and increased the apoptosis ability of CC cells. The migration, proliferation, and apoptosis of CC cells returned to normal levels when they were transfected with miR-186-3p and knocked down with IGF1 simultaneously. These observations indicated that miR-186-3p and IGF1 have opposite effects on CC cells. Thus, miR-186-3p can reduce the incidence of CC by targeting IGF1.

Our research only involved study and verification through in vitro experiments. However, cancer is a complex process. In the future, a deeper understanding will be provided by focusing on the downstream mechanism of miR-186-3p targeting IGF1, and the use of two animal models.

## Conclusions

The data provide the first demonstration that targeting IGF1 by miR-186-3p can regulate CC progression. More specifically, by targeting IGF1, miR-186-3p can inhibit the proliferation and migration of CC cells, and induce apoptosis of CC cells. The data will provide more insights into the progression and treatment of CC.

## Data Availability

The datasets used and/or analyzed during the current study are available from the corresponding author on reasonable request.

## Abbreviations

CC: Cervical cancer
IGF1: Insulin-like growth factor 1
CDK1: Cyclin-dependent kinase 1
HPV: Human papillomavirus
miRNA: MicroRNAs
NC: Negative control
qRT-PCR: Quantitative real-time polymerase chain reaction
UTR: Untranslated region
